# Localisation of Faecal Incontinence Based on History and Physical Examination: A Practical Guide for Neurosurgical Residents

**DOI:** 10.21315/mjms-03-2026-174

**Published:** 2026-04-30

**Authors:** Nik Zulkifli Nik Ab Rahman, Jafri Malin Abdullah, Song Yee Ang, Tharsyini Nair Thilagar, Surin Etican Paramasevan, Shaun Aaron Jeevan, Faris Suhaimi Razali, Saktivadivelu Letchmanan, Yuvaraaj Lechumanan, Keen Chee Kong

**Affiliations:** 1 Department of Neurosciences, School of Medical Sciences, Universiti Sains Malaysia, Health Campus, Kubang Kerian, Kelantan, Malaysia; 2 Brain and Behaviour Cluster, School of Medical Sciences, Universiti Sains Malaysia, Health Campus, Kubang Kerian, Kelantan, Malaysia; 3 Department of Neurosciences and Brain Behaviour Cluster, Hospital Universiti Sains Malaysia, Universiti Sains Malaysia Specialist Hospital, Health Campus, Kubang Kerian, Kelantan, Malaysia

**Keywords:** faecal incontinence, bowel incontinence, neurogenic bowel, bowel control, neurological localisation

## Abstract

Faecal incontinence (FI) is defined as the involuntary loss of stool or flatus and represents an important clinical problem that significantly affects quality of life. Normal continence depends on coordinated function between the rectum, anal sphincters, pelvic floor musculature, and neural pathways involving cortical, brainstem, spinal, and peripheral components. Disruption at any level of this complex system may result in bowel control dysfunction. Clinically, FI may present as urge, passive, or overflow incontinence depending on the underlying pathophysiology. Neurological lesions affecting the frontal cortex, spinal cord, sacral nerve roots, or peripheral nerves may impair voluntary control or disrupt reflex mechanisms of defecation. Careful clinical assessment, including detailed history and neurological examination, is therefore essential for accurate localisation of the underlying lesion and identification of reversible causes. Understanding the neuroanatomy and physiology of bowel continence is important for clinicians, particularly neurosurgeons, when evaluating patients with bowel dysfunction.

## Introduction

Faecal incontinence (FI) refers to the inability to control the passage of stool or gas (1, 2). The severity varies from occasional leakage of stool to complete loss of bowel control (2, 3). Even mild symptoms can significantly impair social functioning and psychological well-being (1, 3). Epidemiological studies estimate that FI affects approximately 7% to 15% of adults, with a higher prevalence in elderly individuals and women (1). Despite its relatively high prevalence, the condition is frequently underreported due to embarrassment and social stigma (1, 3). As a result, many patients do not seek medical attention until symptoms become severe (1, 3). FI may lead to anxiety, depression, and social isolation, further emphasising the importance of early recognition and management (1, 3). A clear understanding of the mechanisms that maintain normal bowel continence is therefore essential for clinicians involved in patient care (2, 4).

## How Normal Continence Works

Faecal continence depends on coordination between the anatomy and the nervous system.

### Rectum and Anorectal Angle

The rectum functions as a compliant reservoir that temporarily stores faecal material. It is capable of accommodating increasing volumes of stool with minimal rise in intraluminal pressure until a threshold volume is reached. Stretch receptors located within the rectal wall detect distension and transmit signals that generate the sensation of rectal fullness and the urge to defecate. The anorectal angle plays an important role in maintaining continence. This angle is formed by the puborectalis muscle, which acts as a sling around the junction between the rectum and the anal canal. At rest, the puborectalis maintains the anorectal angle at approximately 90°, which helps prevent involuntary stool leakage. During defecation, relaxation of the puborectalis muscle straightens the anorectal angle, facilitating the passage of stool (2, 4).

### Internal Anal Sphincter (IAS)

The IAS is composed of smooth muscle and functions as an involuntary sphincter that maintains the majority of resting anal canal pressure. It contributes approximately 70% to 85% of basal anal tone, thereby playing a major role in maintaining continence at rest. Autonomic innervation regulates IAS tone. Sympathetic fibres originating from the thoracolumbar spinal cord promote contraction and help maintain continence, whereas parasympathetic fibres arising from the sacral segments (S2–S4) facilitate relaxation during defecation. Damage to the IAS or its autonomic innervation can reduce resting pressure and lead to passive FI (4).

### External Anal Sphincter (EAS)

The EAS consists of striated muscle and provides voluntary control of defecation. It contributes approximately 15% to 30% of resting anal canal pressure and is particularly important for maintaining continence during sudden increases in intra-abdominal pressure, such as coughing or lifting. Motor innervation of the EAS is provided primarily by the pudendal nerve (S2–S4). Under normal conditions, the sphincter maintains a baseline level of tonic contraction that can be voluntarily increased when necessary to delay defecation. Cortical control, particularly from the medial frontal cortex including the paracentral lobule, enables conscious regulation of sphincter activity (2, 4, 5).

### Neural Control of Defecation

Defecation is regulated through complex interactions between cortical centres, brainstem pathways, spinal reflexes, and peripheral nerves (4–7). Rectal distension activates afferent sensory pathways that transmit signals to the spinal cord and brainstem. A reflex pathway located within the spinal cord coordinates relaxation of the IAS and contraction of the rectum. Higher cortical centres, particularly the frontal cortex and anterior cingulate gyrus, provide voluntary control over this reflex mechanism. These cortical regions allow individuals to suppress defecation until an appropriate time and place. If defecation is delayed, voluntary contraction of the EAS and pelvic floor muscles increases anal canal pressure and prevents stool passage. The autonomic nervous system also plays an important regulatory role. Sympathetic activity promotes stool storage by increasing sphincter tone and reducing colonic motility, whereas parasympathetic activity promotes defecation by facilitating rectal contraction and sphincter relaxation. Together with intrinsic enteric nervous system activity, these mechanisms ensure coordinated bowel function (4, 5).

## Types of FI

### Urge Incontinence

Urge incontinence occurs when patients feel a strong need to defecate but are unable to retain stool long enough (4, 5). This happens when the sphincters or pelvic floor are weak, or when the bowel contracts too hard. For example, injuries to the external sphincter (from childbirth tears or surgeries) weaken control, and conditions with fast diarrhoea or inflammation (like Irritable bowel syndrome or ulcerative colitis) can force stool out despite strong sphincters. On the neural side, if the brain’s inhibition is weak (e.g., early Alzheimer’s or small frontal lesions), the person might feel the urge and try to hold it, but the reflex wins. Patients with urge FI often say they have little warning and a sudden need to find a toilet, with leakage often during loose stools or when coughing or exercising. Asking, “Do you feel a strong urge to defecate but still leak stool?” helps identify urge incontinence.

### Passive Incontinence

Passive incontinence refers to involuntary stool leakage without awareness (4). This means sensory or reflex pathways are damaged. A common cause is nerve damage in the sacral (S2–S4) area: for example, diabetes can hurt sensory nerves to the rectum (blunting awareness) and autonomic nerves to the IAS (weakening resting tone), so stool just leaks. Spinal cord injuries affecting the sacral segments, or pudendal nerve injuries, can also block sensation and reflexes. Patients usually have a very loose anal sphincter tone on exam and cannot hold gas. Severe dementia is another cause: even if nerves are okay, the brain does not notice the stool or forgets to act, so accidents happen. Passive FI is typical in lower motor neuron (LMN) problems of the bowel (like cauda equina syndrome). History clues: patients often describe soiling without warning. Asking, “Do you ever leak stool without feeling the need to go?” points to passive incontinence and suggests checking sphincter tone and sensation.

### Overflow (Seepage) Incontinence

Overflow incontinence occurs when faecal impaction or severe constipation causes leakage around retained stool (5). Here, leakage happens because the rectum is overly full, not because the sphincters or nerves are directly broken. Chronic constipation and impaction are classic causes. The rectum stretches and becomes desensitised, so liquid stool seeps around hard impaction (overflow diarrhoea). Patients may have constant dribbling or underwear staining, but no real urge, and the exam shows a rectum packed with stool. Another form is after a normal bowel movement: a bit of stool leaks out later if evacuation was incomplete. This can happen with a rectal prolapse or rectocele trapping stool. Sometimes a flaccid reflex bowel (as in cauda equina) fills up and then overflows. To detect this, ask: “Do you have to strain a lot or feel like you never empty fully?” or “Do loose stools leak out between normal bowel movements?” If yes, impaction is likely, and treatment focuses on clearing stool (laxatives, enemas, disimpaction) rather than sphincter repair.

## Localisation

Bowel control problems can point to the location of neurological injury or dysfunction (4, 5). Recognition of these patterns is particularly important for neurosurgeons, as bowel dysfunction may represent an early indicator of spinal cord compression or cauda equina syndrome.

### Cortical Lesions (Frontal Lobe)

The medial frontal lobes help voluntarily inhibit bowel and bladder urges (4, 6, 7). Bilateral damage here (from large tumours, haemorrhage, or degenerative disease) causes a “frontal incontinence”: the person feels the need to go but cannot stop it. They often have other frontal signs (changes in personality, reflexes like the grasp reflex, trouble planning, or a wide-based “magnetic” gait as in normal-pressure hydrocephalus). Usually, they are aware of accidents (causing embarrassment). Smaller, one-sided lesions often have milder effects because the other side compensates. Finding incontinence with these signs suggests a brain scan is needed.

### Brainstem/Spinal Cord Lesions (Above Sacral Cord)

Lesions between the brainstem and sacral segments may produce a spastic or reflex neurogenic bowel (4, 5, 8). In this condition, the rectum empties reflexively without voluntary control, often accompanied by constipation because defecation is incomplete. Initially, there may be “spinal shock” with no reflexes, but later reflexes return: anal wink and other reflexes stay or become brisk. Patients often have normal or high resting anal tone, but poor control. They usually need a strict bowel programme (timed suppositories and/or digital stimulation) to empty. On exam, such patients have increased leg reflexes, spasticity, or a sensory level on the torso. If someone has back pain or leg weakness plus new FI, check urgently for a spinal lesion (e.g., epidural compression). A special case is tabes dorsalis (syphilis): the person loses rectal sensation, but motor function remains, leading to unperceived (passive) leakage despite normal sphincters.

### Conus Medullaris/Cauda Equina (Sacral LMN Lesions)

Sacral LMN lesions produce an areflexic bowel with reduced sphincter tone (4, 8). The sacral reflex arc is lost, so both involuntary reflex and voluntary control are gone. The IAS loses tone, and the EAS is denervated, making the anus very loose. Reflexes like the anal wink disappear. The rectum becomes a large reservoir, and stool can overflow continuously. Patients often have severe constipation (huge rectum) with continuous dribbling once pressure builds. This is seen in cauda equina syndrome (large lumbar disc herniation, tumour, etc.), usually with leg pain/weakness and saddle anaesthesia. A conus lesion (at T12–L1) causes more equal (symmetric) loss of bowel/bladder control and saddle numbness, with less leg weakness than cauda. In any sacral LMN lesion, expect passive incontinence (no urge, leaking without warning) and poor evacuation (often needing enemas or manual removal). The anal tone is very low on the exam. Management relies on bowel routines and manual emptying since reflex methods do not work.

### Peripheral Nerve Injuries

Damage to nerves (especially the pudendal nerve) can cause incontinence even if the spine is fine. The pudendal nerve carries motor fibres to the EAS and sensation from the anus. Stretch or compression injuries (often during childbirth, prolonged labour, or forceps delivery) can damage this nerve or the sphincter directly. This can cause immediate or delayed FI. Similarly, chronic straining (from lifelong constipation) can injure these nerves. Diabetes can injure both autonomic (IAS) and somatic (EAS) nerves, causing mixed passive/urge incontinence. In these cases, the exam shows weak squeeze and maybe a missing anal wink, but leg function is normal. Pelvic surgeries (like radical hysterectomy or rectal cancer surgery) and radiation can also damage these nerves or muscles over time, leading to incontinence. Peripheral causes often produce both urge and passive symptoms (weak squeeze and low sensation).

### Sphincter or Pelvic Floor Damage

Direct injury to the muscles can cause incontinence even if the nerves are intact. Childbirth can tear the anal sphincters (3rd or 4th degree tears), leading to immediate or later FI. Anal surgeries (hemorrhoidectomy, fistula repair, or internal sphincterotomy) can weaken the sphincters. If the external sphincter is cut or torn, the person usually has urge incontinence (feeling the need but the muscle cannot hold gas or stool). If the internal sphincter is damaged, resting tone drops and loose stool or mucus can leak. Pelvic organ prolapse (like a rectocele bulging into the vagina, or rectal prolapse) can trap stool or disrupt the angle, causing leakage. A big rectocele can leave stool behind, which then leaks out later. On exam, look for scars, a gaping anus, or visible prolapse with straining, and ask about past births or surgeries.

### Functional or Other Causes

Not all FI comes from nerves or muscles. Severe diarrhoea (from irritable bowel syndrome, inflammatory bowel disease, infections, etc.) can overwhelm a normal sphincter. Loose stools are harder to hold, so controlling diarrhoea often fixes FI. Medications or foods can cause frequent loose stools (laxatives, antibiotics, metformin, high-caffeine or sorbitol foods), so review those. Mobility issues can cause “functional incontinence”: a person may feel the urge but cannot get to the toilet fast enough (common in severe arthritis or after stroke). In such cases, solutions include commodes or scheduled toilet breaks. Dementia can cause FI: the patient may not recognise the urge or remember what to do, so accidents happen (appearing passive). Rarely, psychiatric or behavioural issues (like encopresis in children) can look like incontinence, but these require different treatment. In complicated cases (e.g., spinal cord rehab), a mix of factors can contribute, needing a team approach.

Figures 1 and 2 show the neural pathway for defecation and the functional MRI brain showing areas involved during defecation, respectively.

## History-taking

Taking a detailed history is important in the assessment of FI, as it helps identify the possible cause, severity of symptoms, and the impact on the patient’s quality of life. It also guides further examination and investigations for appropriate management. Important points to ask include the onset and duration of symptoms, nature of incontinence, stool bowel habits, cognition, associated urinary or neurological symptoms, previous obstetric or surgical history, medication use, and the effect of symptoms on daily activities and emotional well-being.

### Onset and Course

When did it start? Suddenly or gradually? Sudden FI, especially with back pain or leg numbness, is an emergency (think spinal cord or cauda equina). Gradual onset in an older person could be neurodegenerative or cumulative damage (like multiple childbirths). Is it constant or intermittent? Accidents only during diarrhoea or after surgery suggest reversible causes. Duration matters: long-standing FI in diabetes could mean neuropathy; stepwise worsening in a younger person suggests multiple sclerosis.

### Nature of Incontinence

Ask if they feel an urge before leaking: “Do you get a warning (like needing to go) or does stool just come out?” Feeling an urge but unable to hold suggests urge incontinence; leaking without awareness suggests passive incontinence. Ask about what leaks: gas, liquid stool, or solid stool? Early loss of only gas or liquid means mild sphincter weakness; loss of formed stool means major dysfunction. Also, ask if the leakage is constant (dribbling) or occasional. Constant staining suggests overflow/incomplete emptying; occasional accidents (e.g., weekly) suggest urge episodes. Find out the frequency and amount of leakage for severity.

### Bowel Habits

Do they have constipation, diarrhoea, or normal stool? Diarrhoea (from irritable bowel syndrome - diarrhoea predominant, infection, inflammatory bowel disease, etc.) often causes urge leaks. Constipation with hard stools can cause overflow leaks. Ask about straining, stool consistency, and sensation of complete emptying. If FI only happens with loose stools, controlling stool consistency might help. Understanding their usual bowel pattern helps separate primary FI from stool problems.

### Cognition and Mobility

Can they recognise the need to go? Have they ever missed the toilet due to not moving fast enough? Dementia patients might not perceive the urge, and mobility-impaired people might sense the need but cannot get to the toilet in time. These cases call for assistance or scheduling, not sphincter surgery. Note if they need help walking or have balance issues.

### Urinary Symptoms

Ask about bladder control. Problems in both bladder and bowel often point to a neurological cause (spinal cord disease, multiple sclerosis [MS], etc.) because they share nerves. For example: “Do you leak urine or have trouble emptying your bladder?” If yes, a central lesion might be likely. If the bladder is fine, think of more local causes. (Diabetes can cause both, and extensive childbirth injuries can sometimes cause both, too.)

### Neurological Symptoms

Ask about leg weakness, numbness, or pain: “Any weakness or numbness in your legs? Back pain? Shooting leg pain?” These suggest spinal issues. Ask about past MS, stroke, or spine injuries. A history of MS is important because it can cause isolated bowel problems. Specifically ask about saddle anaesthesia: “Do you have numbness around the buttocks or genital area, or changes when you wipe after stool?” Saddle anaesthesia is a red-flag for cauda equina or conus medullaris lesions (8). Also ask about sexual function (erection, sensation). In Parkinson’s or similar conditions, ask about constipation and dizziness (signs of autonomic failure).

### Obstetric/Gynecologic History (Women)

Childbirth can tear the anal sphincters (3rd or 4th degree tears), which may result in FI (3). Ask the number of deliveries, any large babies, forceps, episiotomies, or severe tears (3rd or 4th degree). Damage may appear years later (often after menopause, when tissues weaken). Ask if FI started after any delivery or gynecologic surgery. Also, ask about hysterectomy or prolapse surgery. In men, ask about prostate surgery or pelvic radiation, which can affect the pelvic floor and nerves.

### Anorectal History

Ask about surgeries for haemorrhoids, fissures, fistulas, or prolapse. These can cut or weaken sphincters. For example, lateral internal sphincterotomy (fissure surgery) cuts the IAS. Fistulotomy cuts some muscle. Low anterior resection (rectal cancer surgery) often causes a “low anterior resection syndrome” of urgency and incontinence due to loss of the rectal reservoir. Also ask about trauma (e.g., falls, sexual injury) and radiation (pelvic radiotherapy), which can cause late fibrosis and incontinence.

### Medical History

Check for neurological diseases (stroke, Parkinson’s, MS, spinal injury, spina bifida). Metabolic conditions like diabetes (causes neuropathy) or hypothyroidism (causes constipation) matter. Ask if they had any childhood anorectal issues (imperforate anus, Hirschsprung’s), as these can leave lifelong effects. Review medications: laxatives, stool softeners (overuse causes diarrhoea), opioids, iron, antidepressants (cause constipation), and anything causing loose stool (some antibiotics, metformin, vitamins). Note factors like severe obesity or chronic cough (stress leaks).

### Red Flags

Ask about bleeding, weight loss, fever, or severe pain. For instance, blood mixed with stool could mean IBD or cancer; bright blood on toilet paper might be haemorrhoids or fissure. Weight loss or fever raises cancer or infection concerns. Severe anal pain (especially with bowel movements) could be a fissure, causing withholding and overflow. If any red-flag symptoms are present, refer for gastrointestinal workup (e.g., colonoscopy) to rule out cancer or inflammatory disease.

### Psychosocial Impact

Ask how FI affects their life. Many are embarrassed, avoid social activities, or live near toilets. Gentle questions like “How has this changed your daily routine?” can reveal anxiety or depression. Document coping strategies (pads, diet changes). This info helps plan support and justify treatments.

A structured clinical assessment is essential when evaluating patients with FI, as the pattern of symptoms and examination findings may help localise the underlying neurological lesion. Figures 3a, 3b, and 3c summarise a practical approach to history-taking and physical examination in patients presenting with faecal incontinence.

## Physical Examination

Physical examination should include neurological and anorectal assessment (2, 4).

### General/Cognitive

Observe alertness, memory, and gait. A spastic, scissoring gait suggests long-term spinal cord disease; a wide-based, shuffling gait suggests frontal lobe problems (like normalpressure hydrocephalus). Look for frontal release signs (grasp, snout reflex) in older patients with incontinence. Check the spine for scars or defects.

### Abdomen

Check for bladder distension (palpable bladder) in case of urinary retention. Feel for a hard stool mass, especially in the left lower abdomen (sigmoid colon). Listen to bowel sounds (active with diarrhoea, quiet with severe constipation). A palpable faecal mass suggests impaction, causing overflow.

### Perineum Inspection

Have the patient lie on the side. Look for skin irritation or soiling. At rest, the anus should be a closed slit. A wide-open anus or an odd shape (like an anterior “keyhole” defect from old tear) suggests sphincter damage. Look for external haemorrhoids, fissures (tears), or scars. Ask patient to cough or bear down: watch for prolapse of haemorrhoids or rectum, and in women, check for vaginal bulge (cystocele or rectocele).

### Digital Rectal Exam (DRE)

#### Resting Tone

Feel how tight the sphincter is at rest. Normally, it grips the finger. Low tone means an IAS problem or sacral lesion. High tone (rare) might occur if there’s a painful fissure (patient tightens out of pain). Ask if they feel the finger—loss of sensation suggests sacral nerve damage.

#### Squeeze Pressure

Have them squeeze as if holding in stool. You should feel a strong grip from the external sphincter. Weak squeeze means EAS or pudendal nerve injury. No squeeze at all is a severe sign (complete sphincter tear or S2–S4 lesion). Note if the squeeze can be held or if it quickly fades (fatigue).

#### Anal Wink Reflex

Lightly stroke the skin around the anus. The anus should twitch (wink) with each stroke (pudendal nerve reflex). If it does not, suspect a lesion in the S2–S4 reflex arc (like cauda equina or pudendal neuropathy). Anal wink reflex indicates integrity of the S2–S4 reflex arc (6, 7). Upper motor neuron lesions (cord above sacral segments) usually still have the wink.

#### Bulbocavernosus Reflex (BCR)

In men, squeeze the penis head; in women, pinch the clitoris or pull on a catheter. A normal response is an anal squeeze. Absence suggests S2–S4 damage.

#### Perianal Sensation

Test light touch or pinprick around the anus and genitals. Saddle anaesthesia (loss of feeling in the groin/perineum) is a red-flag for cauda equina or conus lesion. Also, check if they feel your finger inside (deep anal pressure sensation).

#### Rectal Vault

Palpate inside for stool: is it full or empty? A lot of hard stool means impaction/overflow. Feel the rectal walls for masses (tumours). In men, press toward the anterior wall to feel the prostate (very enlarged prostate could cause overflow constipation). In women, feel for a rectocele bulge through the front wall if suspecting one. Large internal haemorrhoids feel like soft cushions. If the patient had a prolapse history, you may not feel it unless it is present now.

#### Anal Canal Pathology

Withdraw the finger slightly and feel the full circumference. You might feel a gap or scar (sphincter defect), often anterior in women. You can ask them to squeeze again to feel if one side is weaker. Note any pain (a fissure typically hurts a lot) or firm nodules (could be a fistula tract or tumour).

#### Pelvic Floor Motion

With the finger in place, ask the patient to “bear down.” Normally, the pelvic floor drops a bit, and the sphincter relaxes. Excessive drooping (more than 3 cm) suggests pelvic floor weakness. Then ask them to do a Kegel (squeeze and lift). You should feel the sphincter tighten and move upward. If they cannot, it suggests pelvic floor or nerve issues (but some people just cannot do this well, even if normal).

### Neurologic Exam

After the anorectal examination, a detailed neurological examination of the upper and lower limbs should be performed.

#### Lower Limbs

Test muscle strength (hip, knee, ankle, toes). Look for spasticity (upper motor neuron signs) or flaccidity (LMN signs). Check reflexes: knee (L3–L4) and ankle (S1). Hyperactive reflexes, clonus, and the Babinski sign mean upper motor neuron (cord). Weak or absent reflexes mean a peripheral nerve or root problem. Test leg sensation (touch/vibration/pinprick) and look for a sensory level on the trunk (where sensation changes).

#### Upper Limbs/Cranial Nerves

Usually normal in isolated FI, but a quick check can catch other issues (e.g., MS lesions affecting the face or arms, or signs of Parkinson’s or multiple system atrophy).

#### Coordination and Gait

Watch their walk if they can. A broad-based ataxic gait or positive Romberg suggests a spinal cord dorsal column issue. A Parkinsonian gait (small shuffling steps) might point to Parkinson’s or multiple system atrophy (multiple system atrophy causes early incontinence). If safe, test finger–nose and heel–shin for cerebellar problems.

### Other Tests

In men, check the prostate if not already done. In women, a pelvic exam is performed for prolapse. Check abdominal reflexes (brushing the abdomen should cause muscles to twitch); loss of abdominal reflexes below a level indicates a spinal cord lesion there. Note any clonus in ankles or other pathological reflexes.

## Examination of FI

The clinical examination of patients with FI should be performed in a systematic manner to identify possible neurological, structural, and functional abnormalities. Figures 4a and 4b summarise the stepwise approach to physical and neurological examination in patients presenting with FI.

## Illustrative Clinical Cases

The following cases illustrate the clinical application of the localisation framework in common neurosurgical conditions.

### Case 1: Cauda Equina Syndrome

A 55-year-old man presented with an acute onset low back pain with radicular pain affecting the left leg, associated with progressive weakness of the left lower limb and difficulty passing urine. He also reported involuntary stool leakage without warning, developing over the past one to two weeks. (Figure 5).

On examination, lower limb tone was reduced over the left side. Power was reduced (3/5) in the left lower limb, while the right lower limb was normal. Left knee and ankle reflexes were reduced, with normal reflexes on the right, and plantar responses were downgoing bilaterally.

Sensory examination revealed reduced sensation over the L5 and S1 dermatomes on the left side, with associated saddle anaesthesia involving the perianal region.

On digital rectal examination, there was markedly reduced resting anal tone with poor voluntary squeeze. The anal wink and bulbocavernosus reflexes were absent.

#### Localisation

Findings are consistent with cauda equina syndrome.

#### Type of Incontinence

Passive FI due to loss of sacral reflex and sphincter control.

#### Clinical Relevance

The presence of unilateral radicular symptoms, asymmetric LMN signs, dermatomal sensory loss, and absent sacral reflexes is typical of early cauda equina syndrome.

### Case 2: Conus Medullaris Lesion

A 60-year-old woman presented with a few weeks’ history of FI and urinary urgency. She described not feeling the urge to pass stool well and occasional leakage without warning. There was no significant back pain or radicular symptoms, and she denied any obvious lower limb weakness (Figure 6).

On examination, lower limb tone was normal. Power was full (5/5) in both lower limbs. The knee reflexes were normal, while the ankle reflexes were reduced. Plantar responses were downgoing bilaterally.

Sensory examination showed symmetrical saddle anaesthesia over the perianal region.

On digital rectal examination, there was reduced resting anal tone with a weak voluntary squeeze. The bulbocavernosus reflex was reduced but still present.

#### Localisation

Findings are consistent with a lesion at the conus medullaris.

#### Type of Incontinence

Predominantly passive FI due to impaired sensation and reduced sphincter control.

#### Clinical Relevance

Early bowel and bladder symptoms with symmetrical saddle anaesthesia and preserved lower limb function, together with knee reflexes preserved while ankle reflexes were reduced, suggest a conus medullaris lesion.

### Case 3: Frontal Lobe Lesion

A 70-year-old man with a history of progressive cognitive decline presented with new-onset FI. He reported being aware of the urge to defecate but unable to hold stool until reaching the toilet. Family members also noted personality changes, apathy, and reduced motivation (Figure 7).

On examination, he was alert but demonstrated impaired attention and executive function, with poor verbal fluency and difficulty with set-shifting tasks.

Motor examination showed normal tone and full power (5/5) in both lower limbs. Deep tendon reflexes were normal, and plantar responses were downgoing bilaterally.

Sensory examination was intact in both lower limbs, including the perianal region.

Gait examination showed a broad-based, magnetic gait, with frontal release signs present, including a grasp reflex.

On digital rectal examination, anal tone was preserved with good voluntary squeeze, anal wink and bulbocavernosus reflex were intact.

#### Localisation

Bilateral medial frontal lobe dysfunction.

#### Type of Incontinence

Urge incontinence due to loss of cortical inhibition.

#### Clinical Relevance

The presence of executive dysfunction, frontal features, and preserved sphincter function with intact sacral reflexes suggests a supraspinal (frontal lobe) lesion. Need to rule out normal-pressure hydrocephalus or other frontal lobe pathology.

## Conclusion

Maintenance of faecal continence depends on the coordinated interaction between anorectal anatomy and complex neural control mechanisms (4, 5). Lesions occurring at different levels of this system produce distinct patterns of bowel dysfunction (4, 6–8). The rectum serves as a storage reservoir, while the internal and external anal sphincters, together with the pelvic floor musculature, maintain closure of the anal canal and allow voluntary control of defecation. Neural regulation involves cortical centres, brainstem pathways, spinal cord reflexes, and peripheral nerves. Lesions occurring at different levels of this system produce distinct patterns of bowel dysfunction.

A systematic clinical approach incorporating detailed history, neurological examination, and anorectal assessment is essential for accurate localisation of the underlying pathology. Understanding the neuroanatomical basis of bowel continence enables clinicians to identify the level of neurological involvement and implement appropriate management strategies.

## Figures and Tables

**Figure 1:**
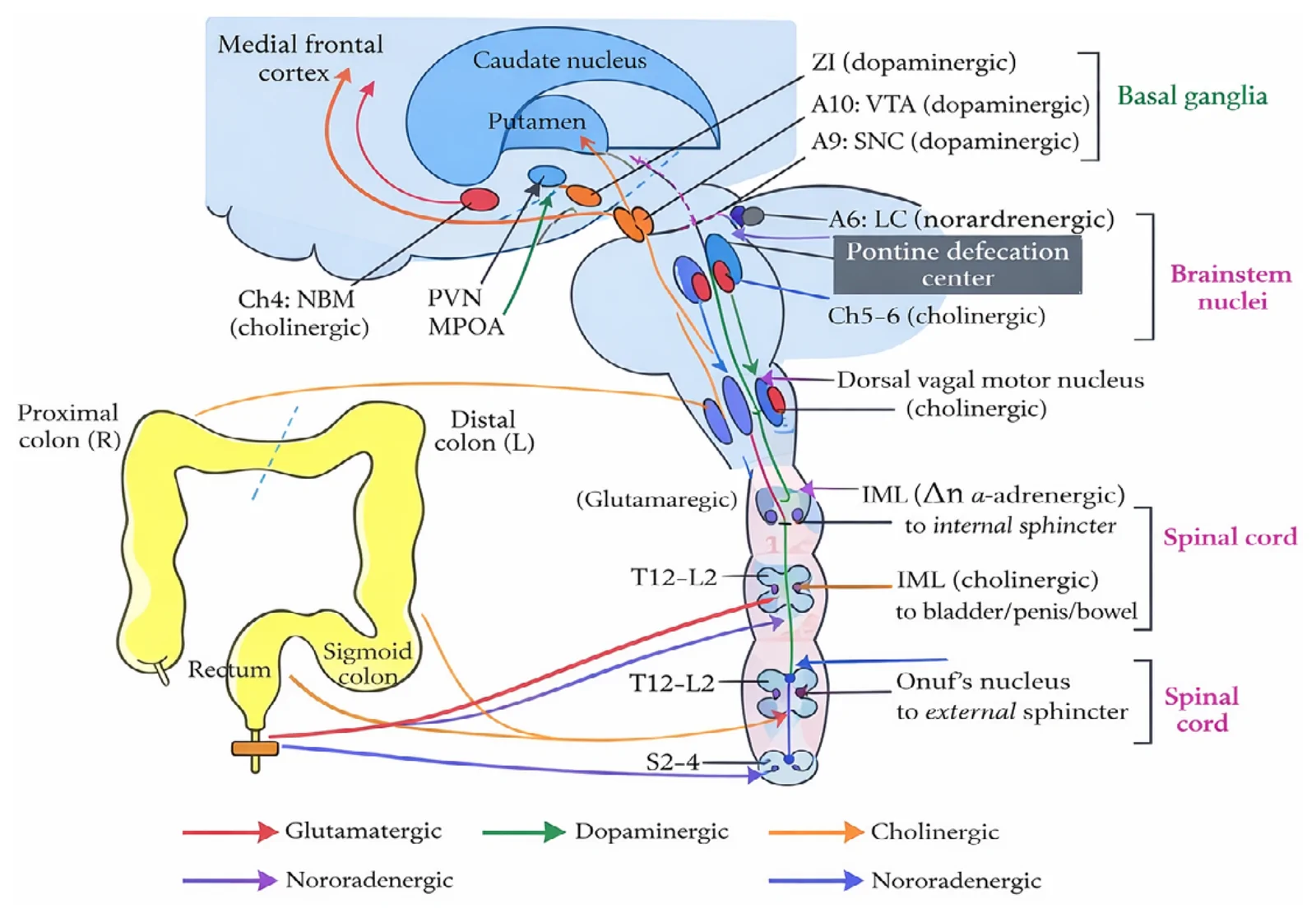
Neural pathway for defecation

**Figure 2:**
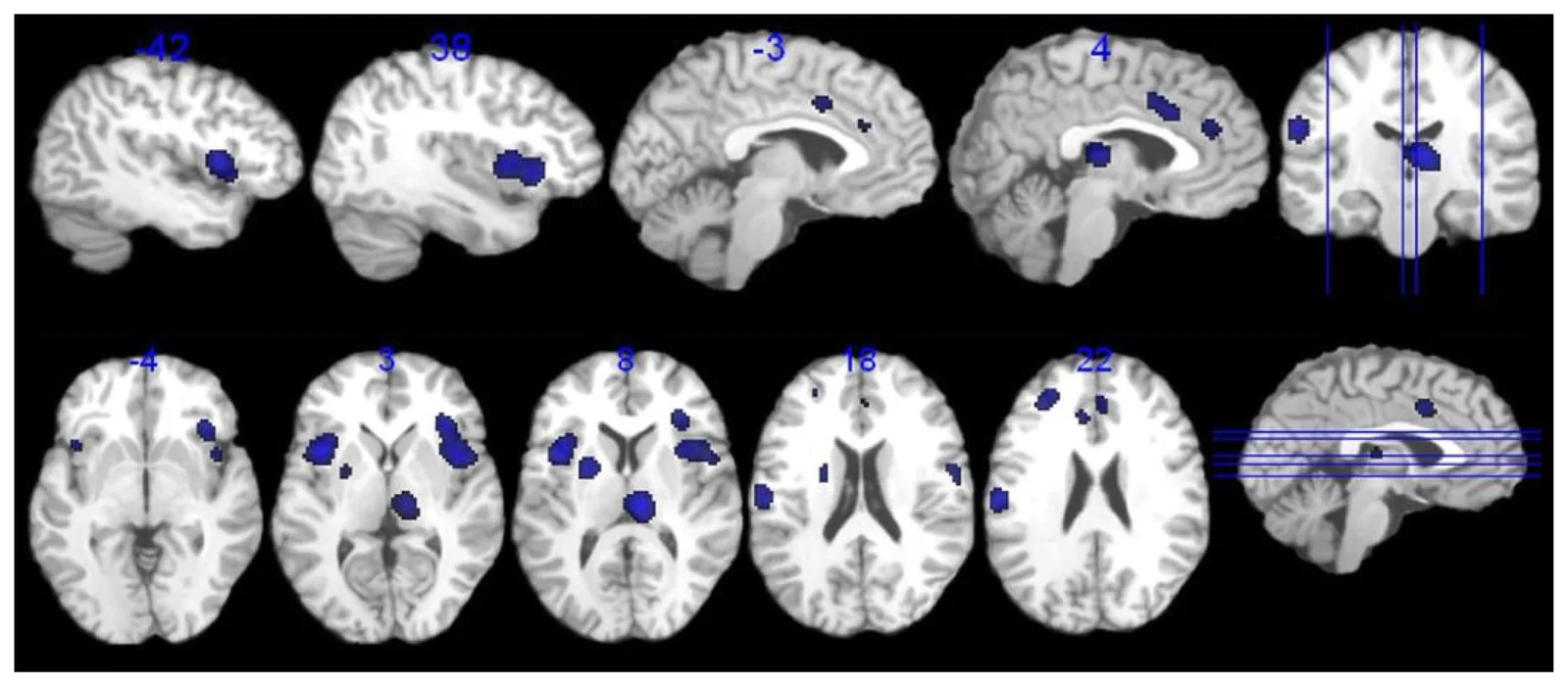
Functional MRI brain showing regions consistently activated in response to supraliminal lower gastrointestinal inflation across healthy control studies, including those associated with visceral sensation Bilateral (B) anterior insula (aINS), B anterior midcingulate cortex (aMCC), and right thalamus; Emotional arousal (right perigenual anterior cingulate gyrus [pACC], BA 32); Regions associated with attention and modulation of arousal (left inferior parietal [BA 40], left lateral [BA 9/46] and right medial prefrontal cortex [BA 9/32]); Consistent activation was seen in the putamen and the post-central gyrus (BA 43)

**Figure 3a:**
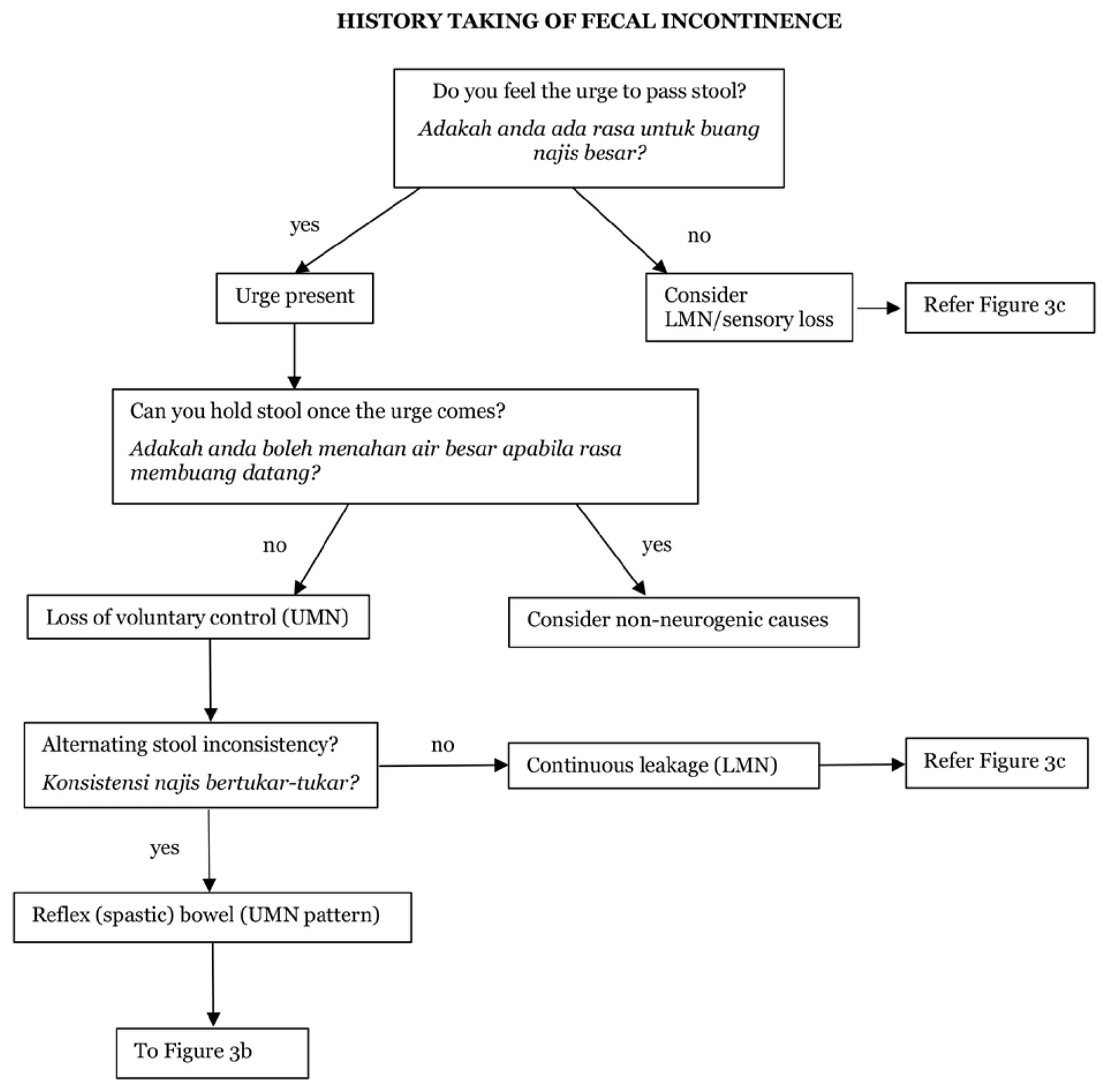
Clinical approach to history-taking and faecal incontinence

**Figure 3b:**
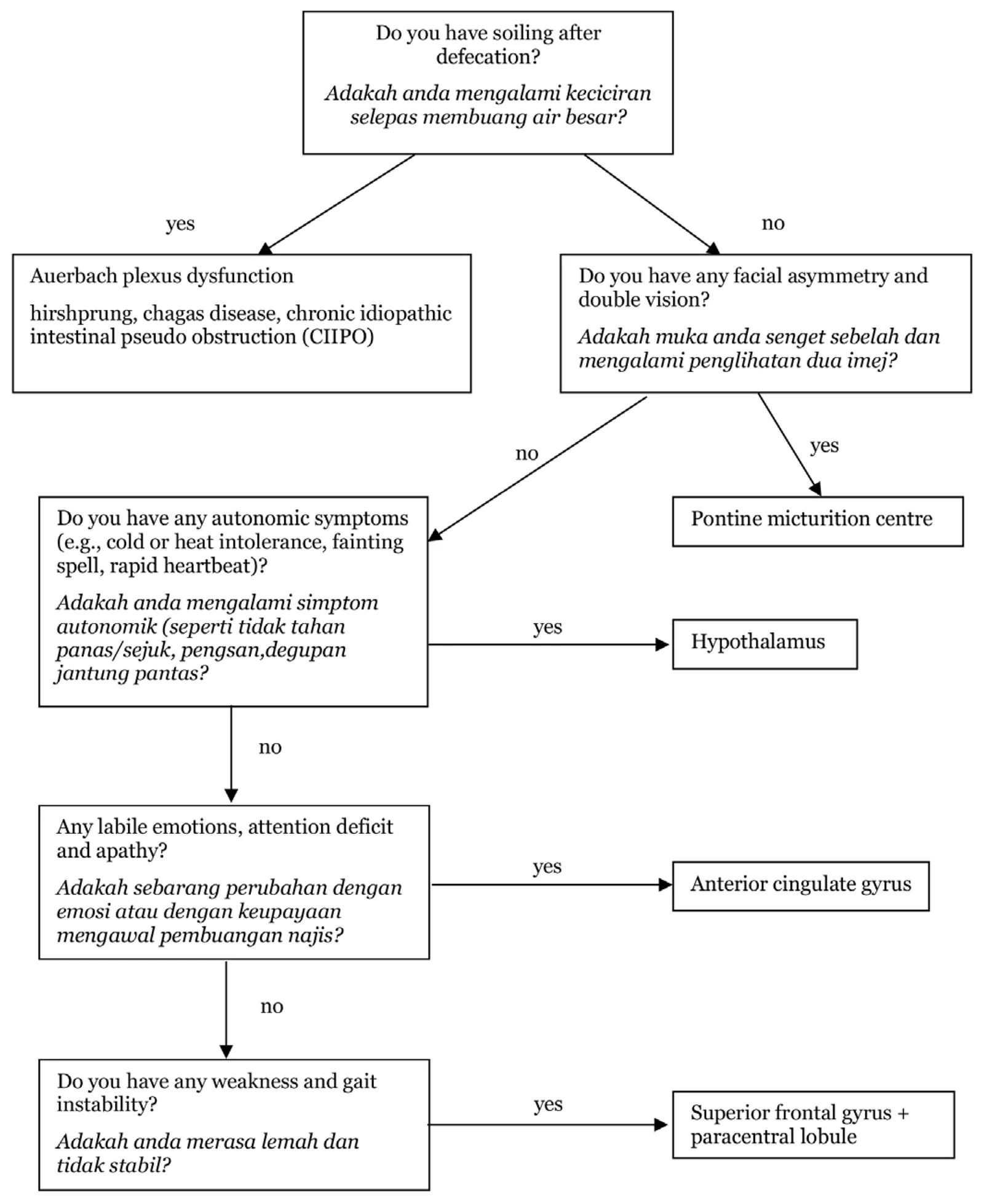
Clinical approach to history-taking and faecal incontinence

**Figure 3c:**
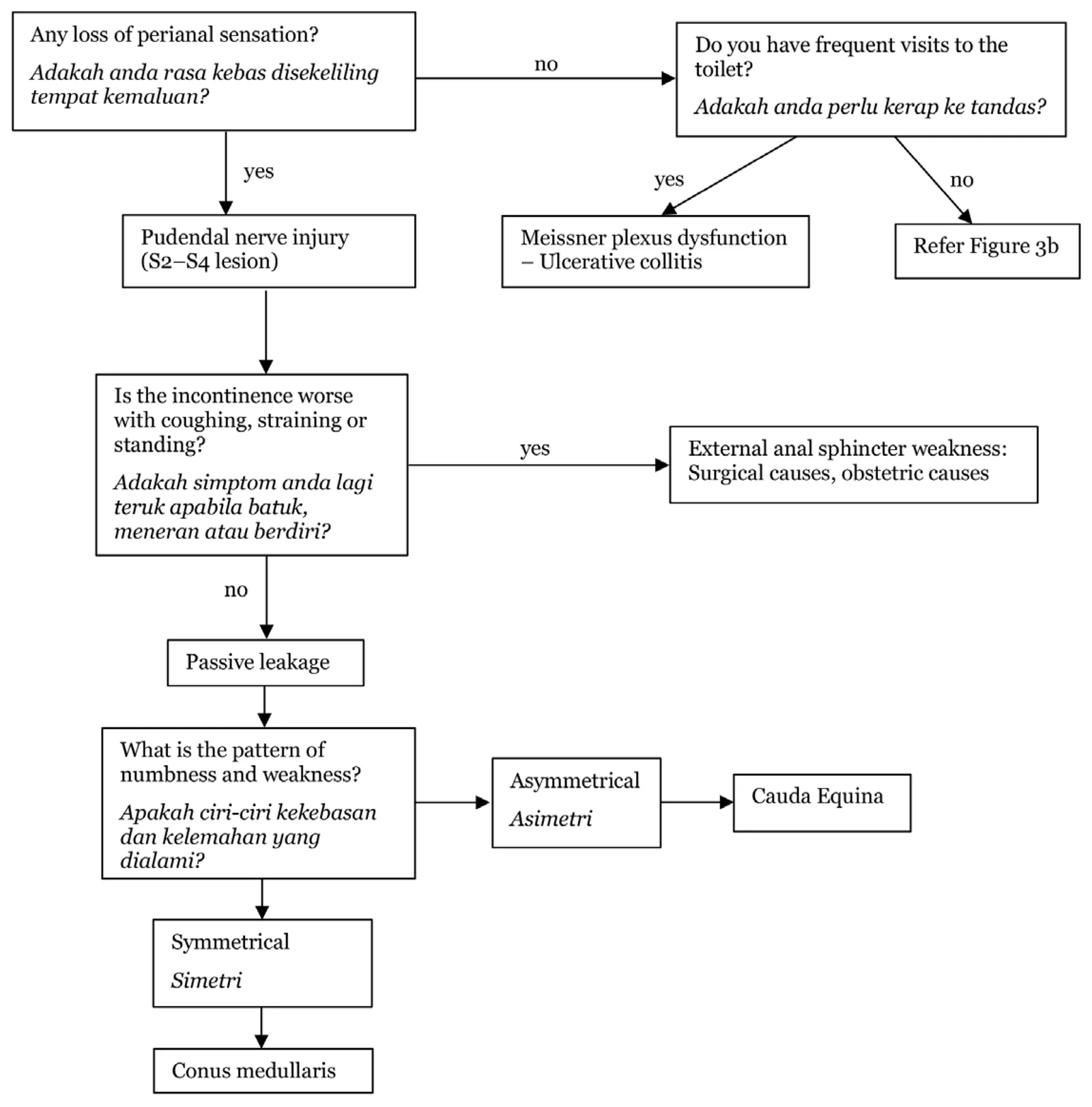
Clinical approach to history-taking and faecal incontinence

**Figure 4a:**
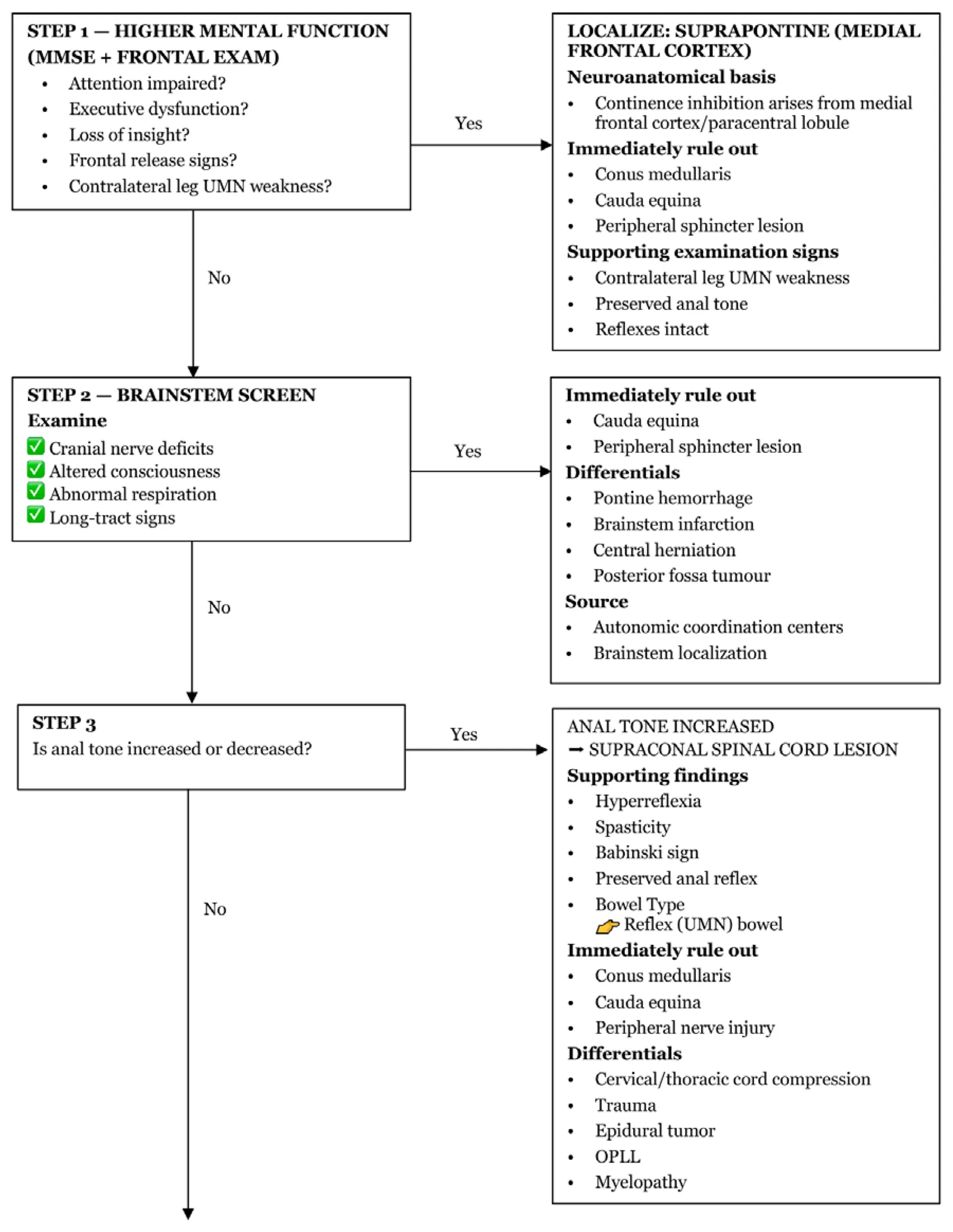
Stepwise neurological examination in patients with faecal incontinence

**Figure 4b:**
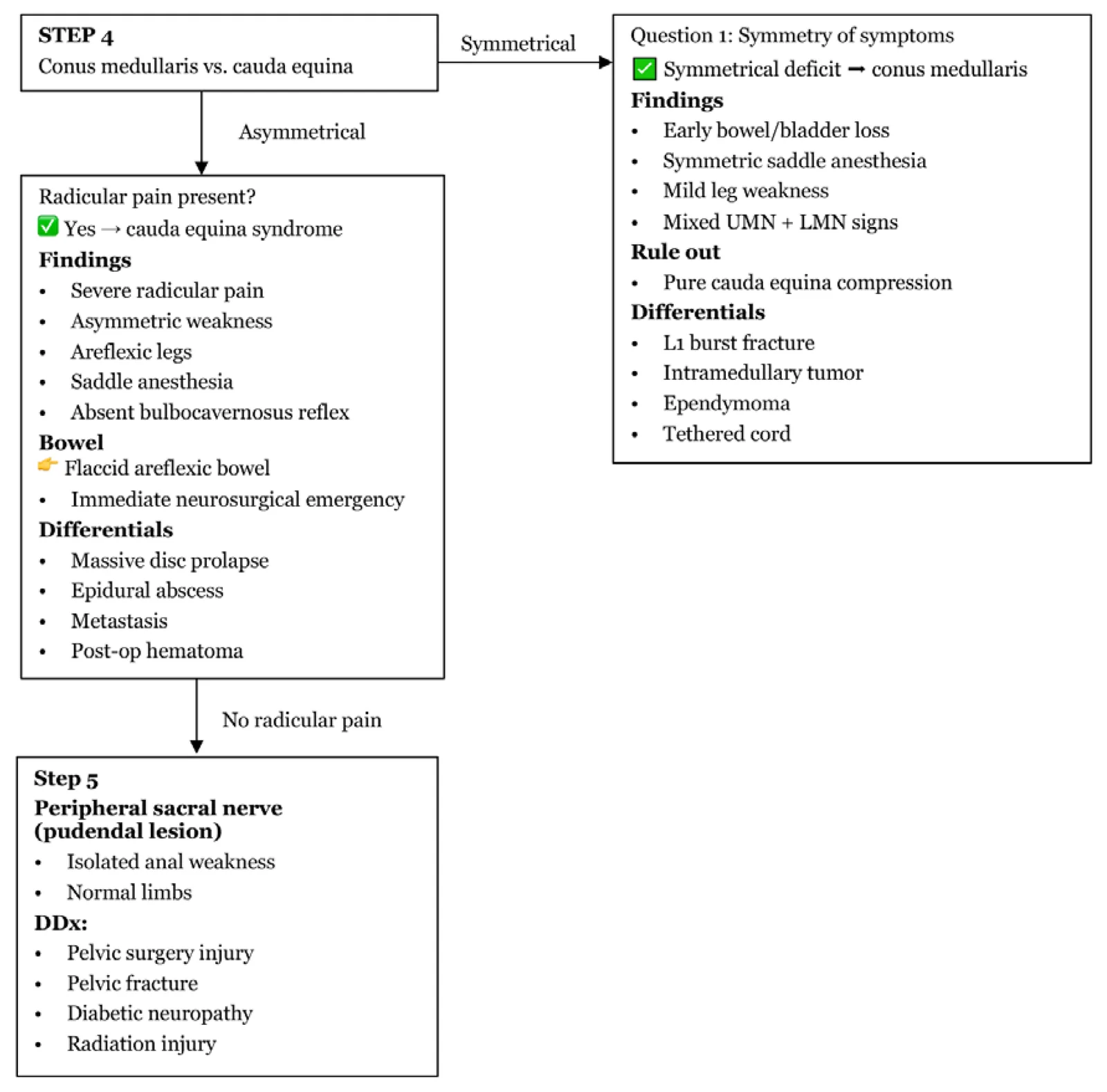
Stepwise neurological examination in patients with faecal incontinence

**Figure 5:**
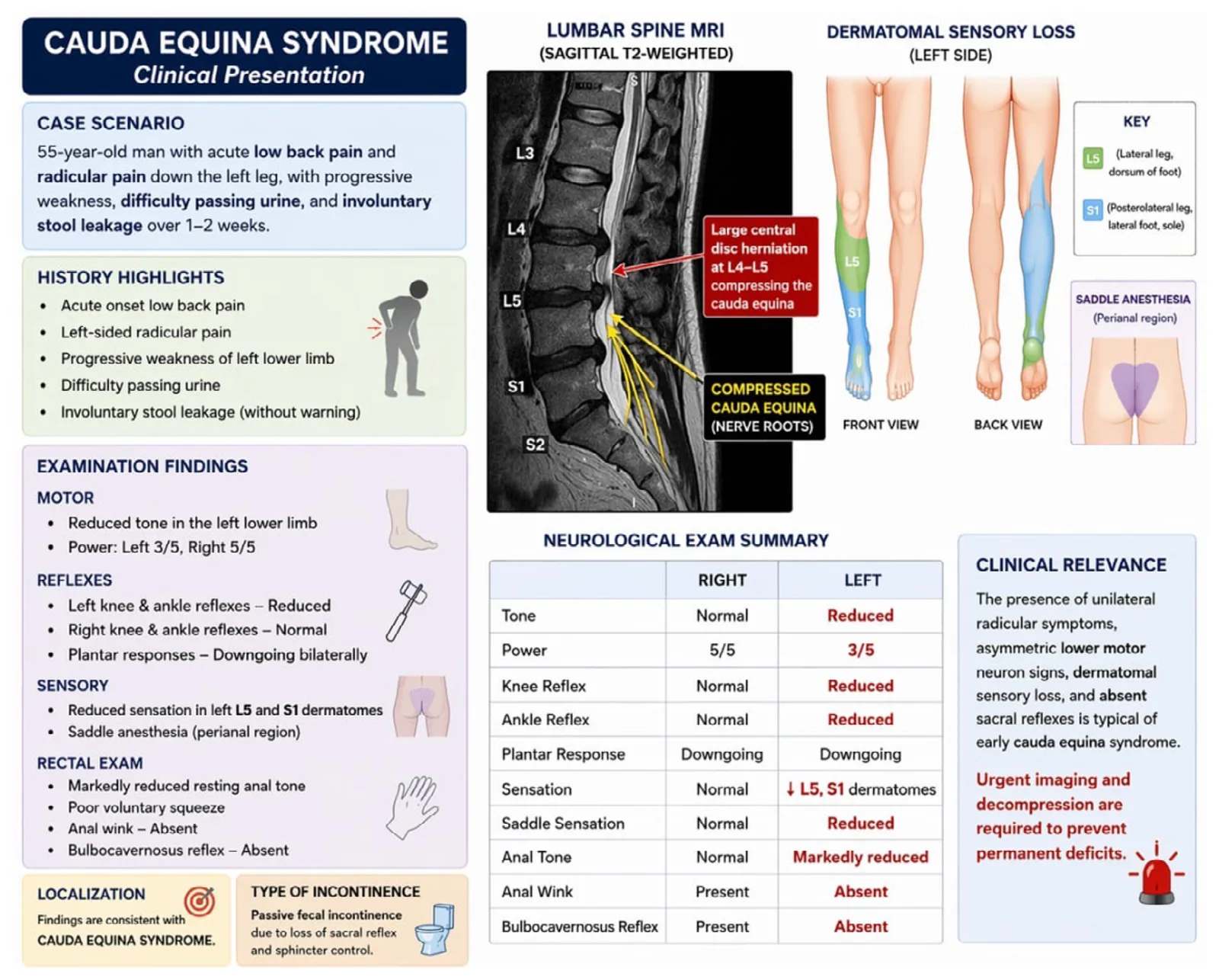
Cauda equina clinical presentation Adapted from Fraser et al. (9)

**Figure 6:**
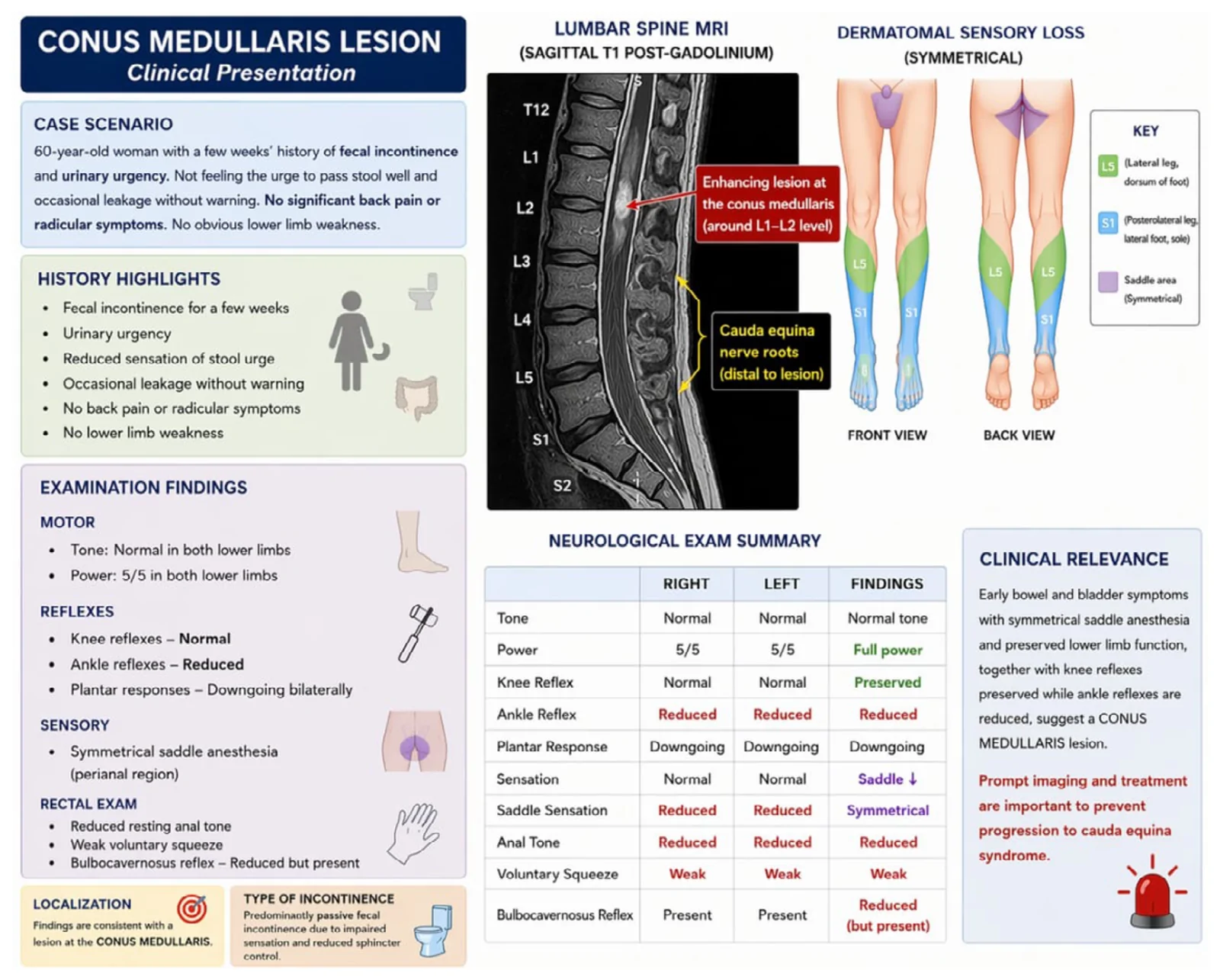
Clinical presentation and localisation features of conus medullaris syndrome Adapted from Brouwers et al. (10) and Rider and Marra (11)

**Figure 7:**
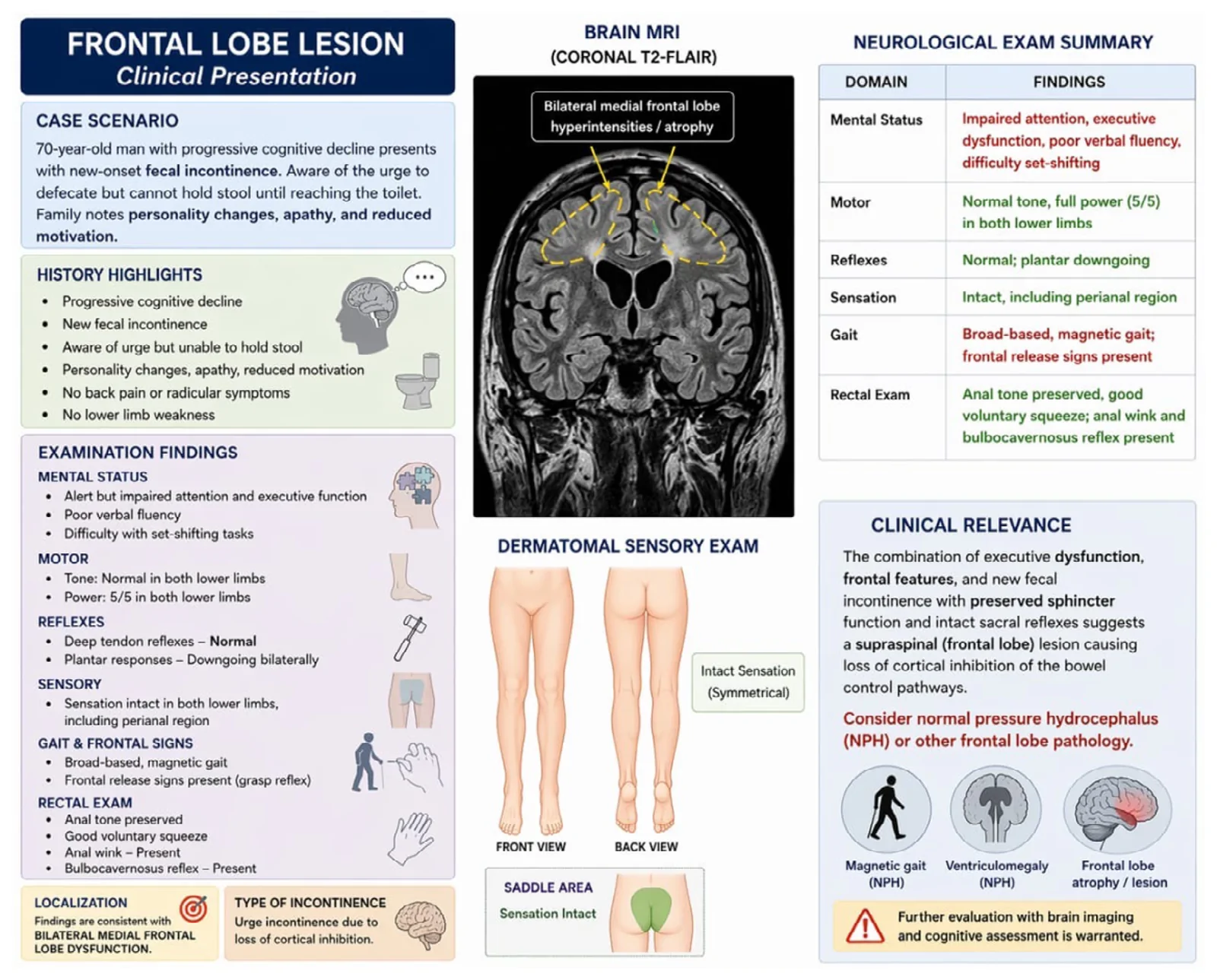
Frontal lobe lesion clinical presentation Adapted from Williams and Malm (12)

## References

[1] Dexter E, Walshaw J, Wynn H, Dimashki S, Leo A, Lindsey I (2024). Faecal incontinence—a comprehensive review. Front Surg.

[2] Rao SSC (2004). Pathophysiology of adult fecal incontinence. Gastroenterology.

[3] Wald A, Bharucha AE, Cosman BC, Whitehead WE (2014). ACG clinical guideline: management of benign anorectal disorders. Am J Gastroenterol.

[4] Remes-Troche JM, Rao SSC (2008). Neurophysiological testing in anorectal disorders. Expert Rev Gastroenterol Hepatol.

[5] Rao SSC (2004). Diagnosis and management of fecal incontinence. Am J Gastroenterol.

[6] Blumenfeld H (2021). Neuroanatomy through clinical cases.

[7] Brazis PW, Masdeu JC, Biller J (2017). Localization in clinical neurology.

[8] Greenberg MS (2023). Handbook of neurosurgery.

[9] Fraser S, Roberts L, Murphy E (2009). Cauda equina syndrome: a literature review of its definition and clinical presentation. Arch Phys Med Rehabil.

[10] Brouwers E, van de Meent H, Curt A, Starremans B, Hosman A, Bartels R (2017). Definitions of traumatic conus medullaris and cauda equina syndrome: a systematic literature review. Spinal Cord.

[11] Rider LS, Marra EM (2023). Cauda Equina and Conus Medullaris Syndromes.

[12] Williams MA, Malm J (2016). Diagnosis and treatment of idiopathic normal pressure hydrocephalus. Continuum (Minneap Minn).

